# Nanoplasmonic biosensors for precision medicine

**DOI:** 10.3389/fchem.2023.1209744

**Published:** 2023-07-06

**Authors:** Yiran Xiao, Zongming Zhang, Shi Yin, Xingyi Ma

**Affiliations:** ^1^ School of Science, Harbin Institute of Technology, Shenzhen, Guangdong, China; ^2^ Biosen International, Jinan, Shandong, China; ^3^ Briteley Institute of Life Sciences, Yantai, Shandong, China

**Keywords:** nanoplasmonic, biosensors, precision medicine, localized surface plasmon resonance, surface-enhanced Raman scattering, lab on a particle, CRISPR/Cas system, cancer diagnosis

## Abstract

Nanoplasmonic biosensors have a huge boost for precision medicine, which allows doctors to better understand diseases at the molecular level and to improve the earlier diagnosis and develop treatment programs. Unlike traditional biosensors, nanoplasmonic biosensors meet the global health industry’s need for low-cost, rapid and portable aspects, while offering multiplexing, high sensitivity and real-time detection. In this review, we describe the common detection schemes used based on localized plasmon resonance (LSPR) and highlight three sensing classes based on LSPR. Then, we present the recent applications of nanoplasmonic in other sensing methods such as isothermal amplification, CRISPR/Cas systems, lab on a chip and enzyme-linked immunosorbent assay. The advantages of nanoplasmonic-based integrated sensing for multiple methods are discussed. Finally, we review the current applications of nanoplasmonic biosensors in precision medicine, such as DNA mutation, vaccine evaluation and drug delivery. The obstacles faced by nanoplasmonic biosensors and the current countermeasures are discussed.

## 1 Introduction

Precision medicine promises to improve health by considering individual variability in genetics, environment and lifestyle (Denny and Collins, 2021). This new medical model provides disease prevention and early diagnosis for patients by combining the latest molecular genetic detecting technologies. The molecular mechanisms of various diseases are revealed through the characterization of genetic material sequences and their products, for example, mutations in the BRCA1 and BRCA2 genes can cause patients to have an increased risk of developing breast and ovarian cancer (da Costa et al., 2020). Therefore, their detection provides a more in-depth and more comprehensive understanding of disease risk. The appropriate detection tools for the diagnosis of individual specific biological indicators help doctors interpret cases more accurately.

Precise diagnosis is a premise for precise treatment, and to meet its requirements, detection tools need high sensitivity, specificity and rapid analysis. Most importantly, it is possible to track and quantify individual information in living cells. Nanoplasmonic sensing is a high spatial resolution optical technique based on localized surface plasmon resonance (LSPR) (Akkilic et al., 2020). Since the scattered light of LSPR is very sensitive to the surrounding media, the binding of trace biochemical molecules to the nanoparticle (NP) surface leads to changes in the local refractive index. This change is reflected in the availability of label-free and real-time presentation on biological macromolecules (Ma et al., 2015). Thus, plasmonic nanoparticles (PNPs) can be used as light scattering probes independent of each other for molecular detection as well as for the analysis of other interactions.

The optical properties of PNPs can be modified by changing their composition, shape and size, and different PNPs can be applied to different situations. Nanoparticles of gold (Au), silver (Ag), and copper (Cu) serve as typical plasmonic materials, which have significant light absorption capabilities in the visible region (Wang et al., 2023). Due to the electron oscillation, different shapes and sizes of nanoplasmonic, such as conventional particles, rods shells and stars, will be special optical phenomena derived from their heterogeneous geometry (Sharifi et al., 2019). By studying the dynamic behavior of nanoplasmonic at the single molecule level in real time, it is important for understanding the biological behavior of living cells and tissues and developing novel nanoplasmonic biosensors.

**FIGURE 1:**
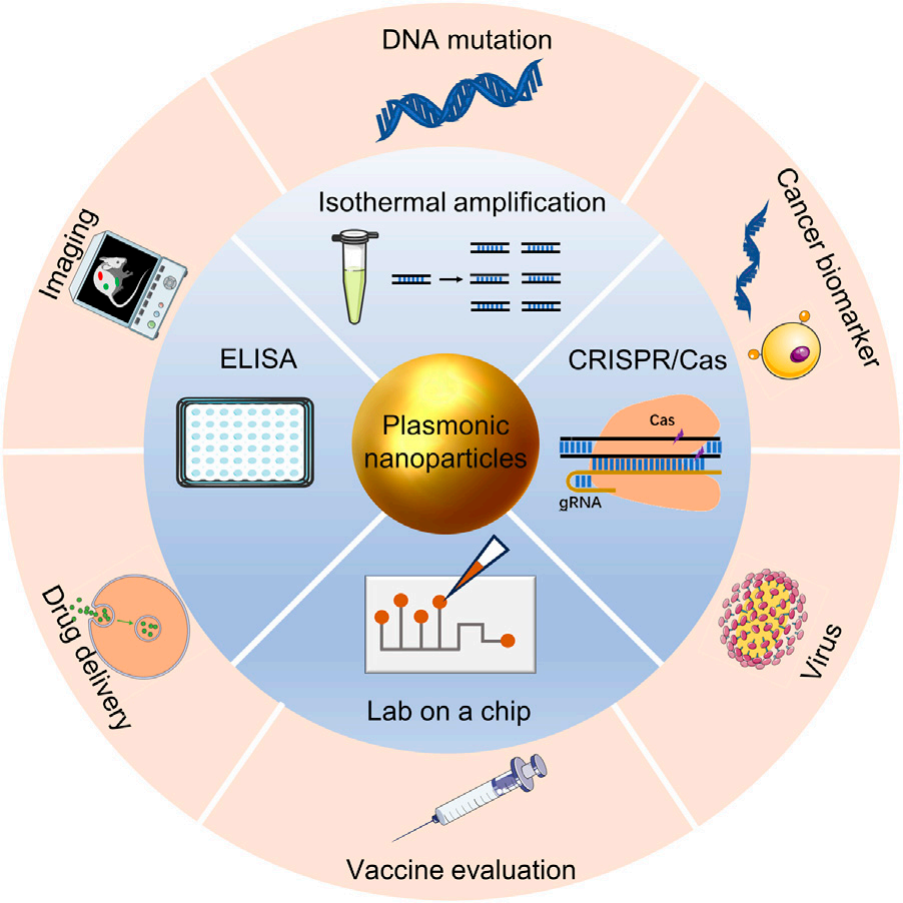
Nanoplasmonic biosensors for precision medicine.

In this review, we describe the principles of nanoplasmonic biosensors, discuss the use of nanoplasmonic biosensors combined other detection tools and research progress in precision medicine in recent years, and provide an outlook on the challenges and future development of nanoplasmonic biosensors (Figure 1).

## 2 Nanoplasmonic sensing

### 2.1 Localized surface plasmon resonance

Precision medicine requires real-time, highly sensitive and *in situ* detection of important substances in living organisms, which poses new requirements for biosensing. In recent years, the rapid development of nanotechnology has provided new opportunities. Nanophotonics-based LSPR sensing enables ultramicroscopic structures, and this combination of the unique optical and electronic properties of nanomaterials has led to a wide range of applications in medical diagnosis and treatment, molecular biology and cell biology (Su et al., 2017).

**FIGURE 2:**
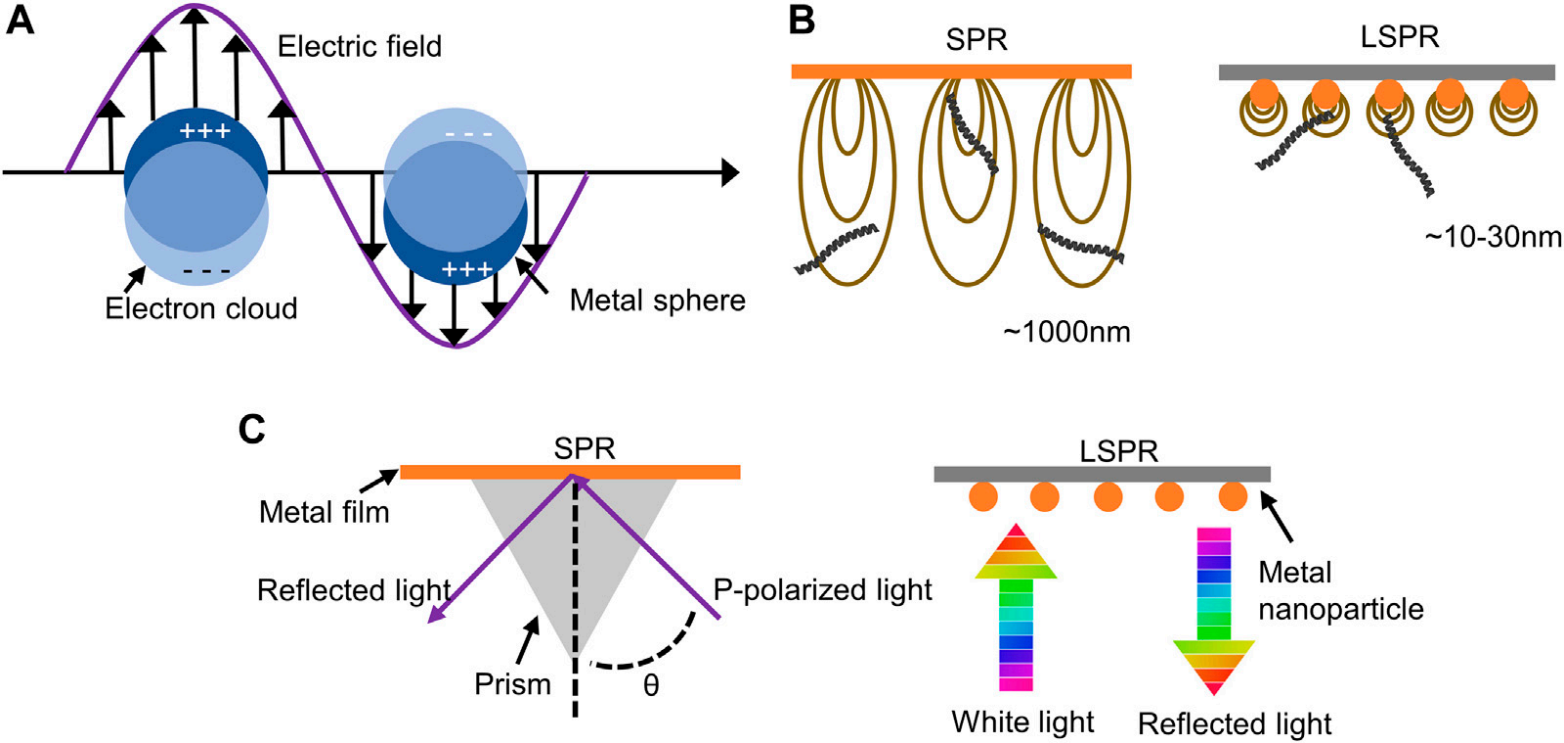
Advantages of LSPR technology compared to SPR. **(A)** Illustration of localized surface plasmon resonance. **(B)** The perception range in LSPR compared to SPR. **(C)** Differences between adaptive optics in LSPR and SPR.

Both surface plasmon resonance (SPR) and LSPR are caused by the interaction of light with metal surfaces. When light travels through a dielectric (such as air or water) to a metal surface and interacts with certain metals (such as Au), collective oscillations of electrons in the conductive band take place at the metal-dielectric interface (Ma and Sim, 2020). If the light is confined to a very small area of the metal nanoparticle surface, it is called LSPR (Figure 2A). The LSPR is much shorter than the evanescent field of SPR. It shows that LSPR can only perceive distances of a few tens of nanometers compared to a micron perception range of the SPR (Jackman et al., 2017; Bousiakou et al., 2020). In other words, in biosensing, due to the large detection range of SPR, SPR may detect biomolecules that are not bound to the sensor surface, resulting in false positives (Figure 2B). Second, since SPR is difficult to satisfy the plasmon resonance condition, the momentum of light needs to be increased by adaptive optics (such as an optical prism) (Figure 2C) (Chauhan and Kumar Singh, 2021). This presents an engineering challenge and increases the complexity of the application.

### 2.2 Types of LSPR-based sensors

Most of the LSPR sensing is performed on gold nanoparticles (AuNPs) or silver nanoparticles (AgNPs). AuNPs and AgNPs are widely used because of the strong fluorescence quenching, resonance and oxidation resistance. (Navas and Soni, 2015; Fai and Kumar, 2021; Kurt et al., 2021). Most importantly, they can easily adsorb biomolecules such as proteins, nucleic acids and other substances, but still retain their optical properties. Stable nanoparticles can be used as sensors to monitor light signals that are triggered by biomolecular interactions through LSPR-sensitive devices.

There are various sensing detection principles based on LSPR, among which the typical types are the use of LSPR absorbance as a readout signal, resonance shifts resulting from local variations in refractive index (refractive index sensing) and field-enhanced characteristic optical radiation (for instance, surface-enhanced Raman scattering, SERS) (Su et al., 2017; Ma and Sim, 2020).

**FIGURE 3:**
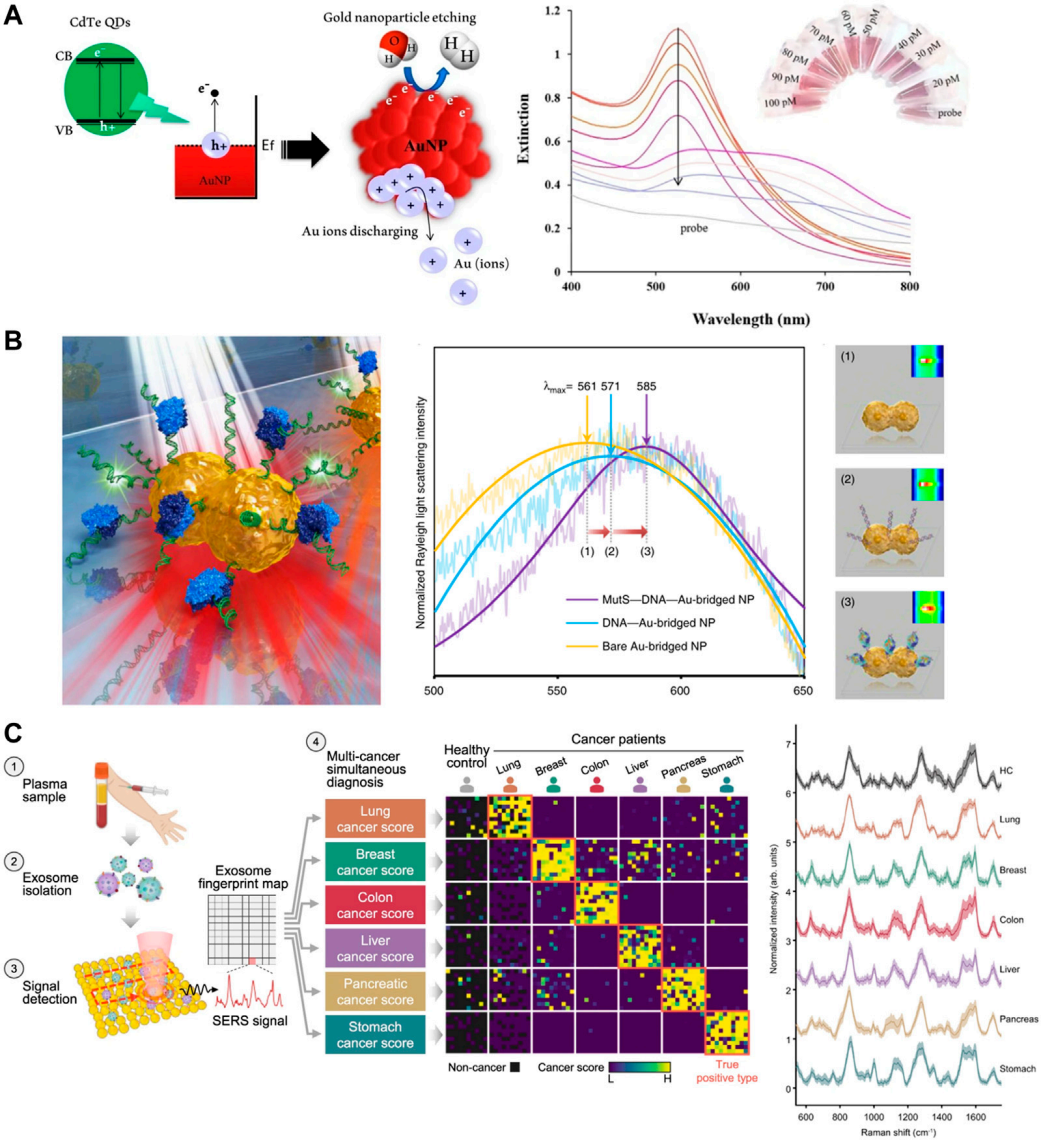
SERS sensors in cancer diagnosis. **(A)** Mechanism of dissolution plasmonic AuNPs with water splitting via hot-electron injection by using CdTe QDs photoinduction, and Extinction band of dsDNA-green QDs complex formed with miR-155 target (right). Reproduced with permission from (Borghei and Hosseini, 2019). **(B)** Schematic illustration of single NP sensing for identifying single point DNA mutations (left) and LSPR λmax shifts (right). Reproduced with permission from (Ma et al., 2019). **(C)** One test-multi cancer using exosome-SERS-AI (left) and representative SERS spectra (right). Reproduced with permission from (Shin et al., 2023).

Nanoparticles utilizing LSPR absorbance as a readout signal are typically small in size, they are more stable in buffer, and shorten the time to detection. The extinction of nanoparticles is usually monitored by chromaticity change or UV-vis spectroscopy (Borghei and Hosseini, 2019). reported a naked eye reading method for the detection of miRNA derived from CdTe QDs photoinduced LSPR solubilized gold nanoparticles (Figure 3A). CdTe QDs brought effects on the extinction bands of AuNPs, and the association of various concentrations of miR-155 and CdTe QDs produced different extinction bands on AuNPs (Kumar et al., 2021). reported a sensitive fiber optic LSPR-based biosensor for the detection of *Shigella*. They utilized coatings of nanomaterials and molybdenum disulfide (MoS_2_) to help excite localized plasma excitations.

According to the Mie theory, the plasma resonance frequency of noble metal nanoparticles frequency is closely related to the refractive index of the surrounding medium (Su et al., 2017). The scattering spectra will be shifted with refractive index changes. Nucleic acid sensing by scattering signal change is much larger than that by absorbance change. The size of AuNPs and AgNPs that are sensed by changes in scattering signal is usually larger than those that are detected by changes in absorbance. This is due to the fact that the nanoparticles need to be sufficiently energetic so that the light scattering signal of each particle can be observed under dark field microscopy (DFM) (Ma et al., 2019). reported a method for rapid identification of point mutations by a single bridge-like AuNP sensor synthesized by DNA guidance (Figure 3B). This high refractive index sensitive biosensor is capable of detecting protein-DNA interactions and detecting single point DNA mutations (Funari et al., 2020). developed an optical microfluidic sensing platform. Gold nanospikes in this platform were fabricated using an electrodeposition method to detect a certain of antibodies specific for SARS-CoV-2 spike protein in human plasma within 30 min. The LSPR wavelength peak shift of gold nanospikes varies with the concentration of antibodies at different targets, which is attributed to local refractive index changes due to antigen-antibody binding. In the same year (Versiani et al., 2020), carried out a nanosensor based on LSPR, which is able to differentiate serologically between dengue and Zika infections. Readings can be obtained in the ELISA-plate spectrophotometer without the need for specific equipment. In recent years, researchers have improved the refractive index sensitivity by improving the size and morphology of gold nanoparticles, and are gradually moving toward miniaturization in terms of experimental setup.

Since Raman analysis cannot detect molecules in ultra-low concentration solutions, surface-enhanced Raman was developed to improve the sensitivity of Raman detection (Zhang et al., 2019) (Balderas-Valadez et al., 2018). When the LSPR spectrum matches the absorption wavelength of the adsorbed molecules, some biosensors can detect Raman signals caused by plasma field enhancement effects, in addition to the signal of the intrinsic LSPR of the plasma. The capability of SERS to enhance the Raman signal by several magnitudes through plasma excitation has led to great scientific interest in knowing the foundation of this improvement (Langer et al., 2020). SERS has been used in various ways for cancer diagnosis. Many research groups have attempted to use SERS in medical diagnostics to detect cancer biomolecules in blood, saliva and urine (Constantinou et al., 2022). (Moothanchery et al., 2022) group developed a fast single peak Raman technique for the diagnosis of epithelial ovarian cancer by haptoglobin, prognostic biomarkers. Haptoglobin concentration in ovarian cyst fluid can be tested and quantified using an *in vitro* based on Raman spectroscopy. Instead of using plasma nanoparticles to enhance the intrinsically weak Raman signal as described previously, this system is quantified by the pure Raman signal intensity of the TMB. Still, it can be seen that the development of low-cost and portable systems is the direction of SERS device development. However, logical multiplex assays with more than three biomarkers remain challenging (Gong et al., 2015). (Lin et al., 2021a) developed a 0.3 cm diameter nanogel matrix which can enhance and stabilize the Raman signal further. This nanogel substrate can capture SERS nanoparticles corresponding to the proteolytic activity of matrix metalloproteinases (MMP), assisting doctors to obtain low concentrations of target MMP to guide subsequent treatment. And more recently, SERS has been further developed in conjunction with AI for cancer diagnosis (Shin et al., 2023). demonstrated a liquid biopsy method combining AI and SERS to diagnose 6 early cancers through label-free analysis of plasma exosomes (Figure 3C). Recent papers have emphasized the need to continue the performance exploration not only in the structure of metallic nanomaterials, but also to focus on the integration, miniaturization and cost reduction of sensors for practical applications. In this regard, disposable, stand-alone integrated sensing’s are perhaps the most practical.

## 3 Integration of nanoplasmonic with other methods

Local enhancement of optical fields displayed in metallic nanostructures has been used in a large number of research areas. In single molecule biosensing, PNP is one of the most researched label-free platforms with high sensitivity (Akkilic et al., 2020). Not only that, with the advancement of nano-plasmas, more biological diagnostics are integrated, greatly facilitating the development of crossover to different subjects.

### 3.1 Isothermal amplification

Mainstream isothermal amplification techniques include loop-mediated isothermal amplification (LAMP), recombinase polymerase amplification (RPA) and helicase-dependent amplification (HDA), etc (Zhao et al., 2015). The entire process of isothermal amplification is always at one temperature, and rapid amplification of nucleic acids can be achieved by designing the appropriate specific primers and adding active enzymes. To facilitate and enhance their performance, various nanomaterials have been introduced in isothermal nucleic acid amplification, mainly for reaction enhancers, signal generation/amplification or surface loading carriers (Zhang et al., 2022).

**FIGURE 4:**
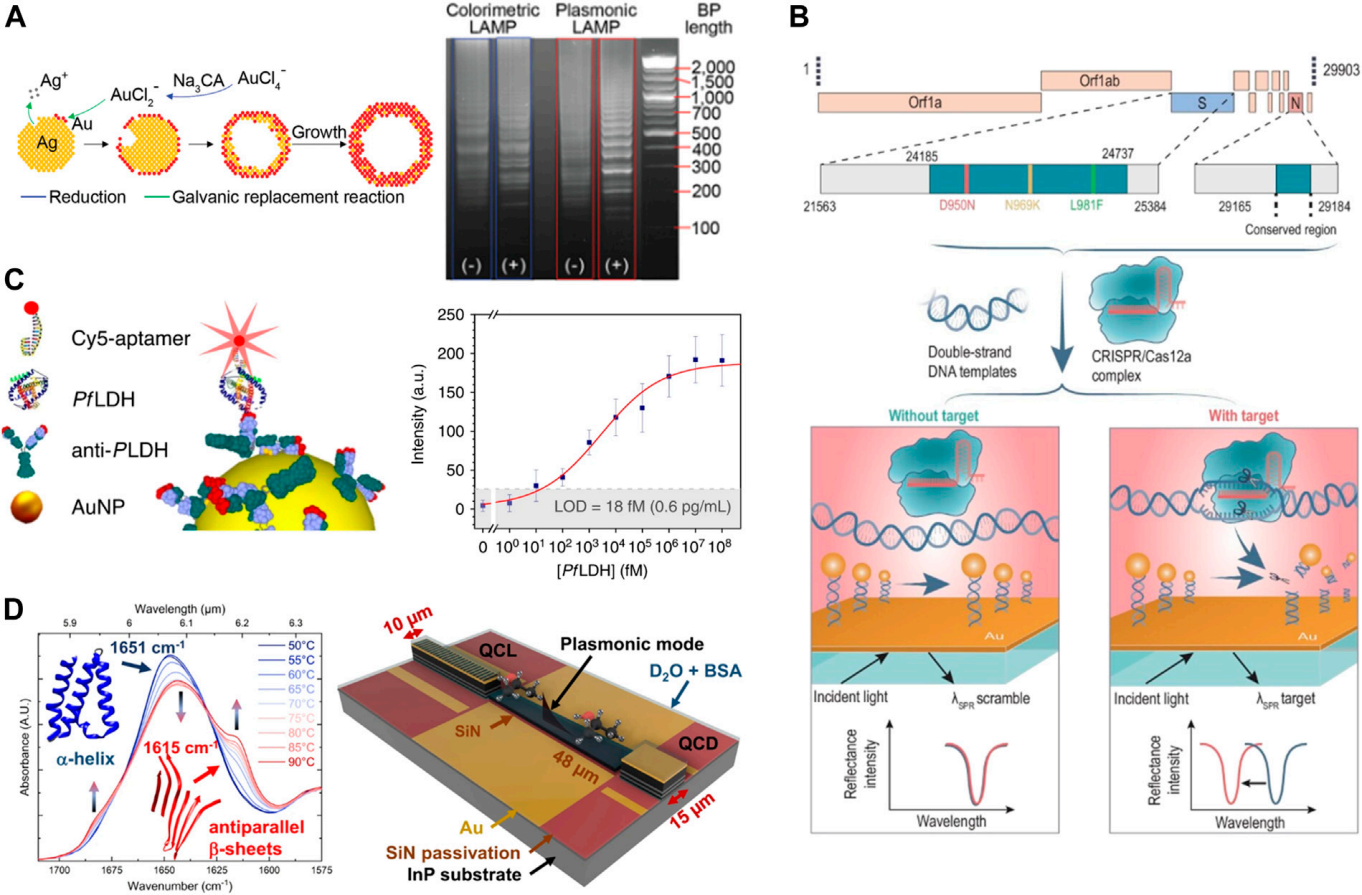
Nanoplasmonic integration strategy. **(A)** Schematic illustration of the Au-Ag shells growth based on galvanic replacement reaction (left)and gel images of plasmonic LAMP (right). Reproduced with permission from (Ye et al., 2022). **(B)** Scheme of MOPCS. Reproduced with permission from (Chen et al., 2022). **(C)** Sketch of the Ab-PfLDH-aptamer sandwich scheme (left) and calibration curve of the immunoassay for *Pf*LDH (right). Reproduced with permission from (Minopoli et al., 2020). **(D)** FTIR spectrum of bovine serum albumin (BSA) (left) and schematic of the QCLD device (right). Reproduced with permission from (Hinkov et al., 2022).

Alafeef et al., 2021 reported a stepwise protocol for rapid and naked-eye molecular diagnosis of COVID-19 with RNA-free extraction nano-colorimetric testing. The binding of ASO, which is specific for the N-gene of SARS-CoV-2, to its target sequence leads to the aggregation of plasmonic AuNPs. Such high-specific aggregation processes resulted in an alteration of the plasmonic response of the nanoparticles. The following year (Ye et al., 2022), reported a powerful sensing method based on DNA hybridization of LAMP amplicons for nucleic acid detection, called Plasmonic LAMP. Au-Ag alloy nanoshells are developed as plasma sensors and display molecular weight standard patterns in gel images (Figure 4A). However, RPA exhibits a faster detection compared to the 75 min reaction time of the LAMP method described above (Woo et al., 2021). developed a plasmonic isothermal RPA array chip that can accomplish fast multiplex molecular detection. The 3D plasmonic substrates comprising AuNPs on intensive gold nanopillars show highly intensified iso-excited enhanced fluorescence of long RPA products.

The application of nanoplasmonic in isothermal amplification is extensive and effective. Because nanoplasmonic significantly increases the sensitivity to isothermal amplification. But traditional isothermal amplification requires purification of nucleic acids and gel electrophoresis, and although the amplification time is shortened, the results show that it takes a significant amount of time. Researchers expected to take advantage of the fast speed of isothermal amplification by combining it with nanoplasmonic and using fluorescence microscopy for simple measurements. However, the overflow of nucleic acids during isothermal amplification and the lack of the complex laboratory equipment remain pressing issues.

### 3.2 CRISPR/Cas system

Recent advances in Clustered Regularly Interspaced Short Palindromic Repeats (CRISPR) and CRISPR associated (Cas) enzymes have exposed the distinctive incidental DNase activities of Cas (Zetsche et al., 2015; Chen et al., 2018). The widely known mechanism of CRISPR technology is the combination of a Cas enzyme with a guide RNA (gRNA). This complex can recognize and cleave site-specific DNA sequences by protospacer-adjacent motif (PAM). The rich variety and diverse properties of CRISPR/Cas systems make them important not only for gene editing, but also show great potential for single-molecule sensing.

In recent years, although different methods for detecting DNA mutations such as SPR and SERS have also been developed, these methods require expensive equipment and do not allow for point of care (POC) applications (Li et al., 2014; Moitra et al., 2022). (Zhou et al., 2022) developed a CRISPR/Cas9-based visual colorimetric platform that specifically detects all single-base mutations. The visual effect was further enhanced by using HRP-gold nanoparticles complex (hGNPs) and biotin modified probes (Bioprobe) to hybridize with RCA products on top of magnetic separation. Similarly, in the same year (Chen et al., 2022), demonstrated a Methodologies of Photonic CRISPR Sensing (MOPCS) for rapid and specific diagnosis of the Omicron variant of SARS-CoV-2 (Figure 4B). The application of this single-base mutation recognition capability highlights the potential for subspecies precision detection applications, is a novel finding, and may replace PCR in future large-scale virus screening.

Besides that, the nanoplasmonic also plays a role in virus detection by enhancing color development (Li et al., 2019), described a new plasma-based CRISPR Cas12a assay for colorimetric detection of red-blotch infection. This sensing strategy generates a fast and specific colorimetric signal for nucleic acid amplicons by combining Cas12a′s unique targeting-induced single-stranded DNase activity with plasma coupling of DNA-functionalized AuNPs. However, in tumor therapy, the direct combination of SHP2 and Elaiophylin was further confirmed by SPR (Li et al., 2022). This contributes to a rational treatment strategy for ovarian cancer (Tao et al., 2022). synthesized multi-branched gold nanocomposites that not only have significant plasmon resonance in the NIR-II window but also control the delivery of CRISPR-Cas9 for synergistic gene photothermal therapy.

Nanoplasmonic is integrated with CRISPR may be the future trend of development. Firstly, compared to the traditional CRISPR strategy of signal amplification with the help of amplification, which is prone to aerosol contamination, nano-plasma combination achieves enhanced sensitivity under no amplification by a different way. Secondly, traditional nano-plasma sensing usually requires the use of precision and complex instruments, while CRISPR systems have great potential for visualization and *in situ* detection. With high sensitivity and accuracy, the characteristics of both are well complemented. It is exciting to note that in addition to diagnostic advances, the synergy of nanoplasmonic and Cas-led gene editing in therapeutic approaches has also yielded good results, which is driving the development of precision medicine.

### 3.3 ELISA

Enzyme-linked immunosorbent assay (ELISA) is a comprehensive technique that combines the immune reaction of antigens and antibodies with the efficient catalytic reaction of enzymes. A common feature of nanomaterials is the high specific surface, which has enabled the immobilization of probes and enhanced detection performance through increased sensitivity (Li et al., 2020). Exploring nanoplasmonic strategies for ultrasensitive detection of protein biomarkers appears to be more challenging than DNA detection.

Luan et al., 2020 reported that plasmonic nanoscale structures can be used as “add-on” tags for various bioassays, improving their signal-to-noise ratio and variable range without changing their workflow and reading devices. In the same year (Minopoli et al., 2020), described an isoexcite-enhanced fluorescent immunosensor to detect *Plasmodium falciparum* lactate dehydrogenase (*Pf*LDH), specifically and ultrasensitively in whole blood (Figure 4C). The biosensor achieves a detection limit of <1 pg/mL (<30 fM) without any sample pretreatment and has very high specificity. Also, (Li et al., 2020), designed a plasma nanoplatform with a catalytic hairpin assembly (CHA) amplification reaction and increased detection limit to 1.0 × 10–^4^ pg/mL In the early diagnosis of hepatitis C virus (HCV) infection, the detection sensitivity was much better than that of commercial ELISA kits. And the 83.3% positivity rate of the plasmonic nanoplatform was higher than the 53.3% of ELISA. Plasmonic nanostructures are candidates for extending fluorescence detection limits to femtomolar levels and beyond.

Naked-eye detection, without any optical readout device, is another advantage of this sensing approach, which significantly reduces the cost of analysis and makes it feasible in resource-limited settings. Although the emerging plasmonic ELISA is a candidate diagnostic due to its unprecedented sensitivity and ease of handling, unimodal colorimetric readouts that rely primarily on the monodisperse or aggregated state of Au or Ag nanoparticles remain limited in clinical applications due to uncertain experimental and environmental factors leading to poor accuracy.

The future direction of nanoplasmonic biosensors relies on fast, sensitive, and fielded strategies that are not only useful for achieving single-molecule sensitivity in vitro bioassays and revealing molecular interactions within organisms, but are also suitable for large-scale applications. This requires the cross-application of multiple technologies and continuous improvement of detection devices to complement the obvious shortcomings of biosensors, thus showing greater potential for integration, portability, and standardization in nanoplasmonic biosensors. Surprisingly, with the advancement of biocompatible and long-term stable nanomaterials, tracking single target molecules will finally open new paths for precision diagnostics and therapeutics.

### 3.4 Lab on a chip

A microfluidic chip is one that manipulates or processes small amounts of fluid through channels of tens to hundreds of microns in size, and is also known as a lab on a chip (LOC). In recent years, advances in LOC technology have facilitated advances in miniaturized bioanalytical equipment (Whitesides, 2006; Liao et al., 2018). (Hinkov et al., 2022) presented a fully monolithic integrated mid-IR sensor which integrates all these functions into a miniaturized device (Figure 4D). By combining a laser, interaction zone, and detector on a single chip, and by using plasmonic waveguides that avoid the diffraction limitations typical of conventional chip-scale photonic systems. They achieved a next-generation fast liquid sensor of fingertip size (<5 × 5 mm^2^) next-generation fast liquid sensor. However, due to the oversimplified nature of most optical architectures, miniaturized systems are usually far less capable than equivalent systems in desktop labs (Tua et al., 2023) developed a compact plasmonic “rainbow” chip for fast and accurate dual-function spectroscopic sensing that can outperform conventional portable spectrometers under certain conditions. The system has the potential to be integrated with smartphones and LOC systems to develop *in situ* analysis applications.

Although LOC has made substantial progress in improving detection throughput, reducing cost and time, and simplifying operation, the improvement of sensitivity in the integration of nanoplasmonic on microfluidic chips is still not negligible (Garcia-Lojo et al., 2020). produced SERS microfluidic chips by integrating plasmonic supercrystals within microfluidic channels for label-free and ultrasensitive detection (Wang et al., 2021). proposed the integration of nanorod arrays on microfluidic chips to be used for rapid and sensitive flow immunoassays of physiologically related macromolecules. Dense arrays of Au nanorods can be easily prepared by one-step oblique angle deposition, thus eliminating the requirement for advanced lithography methods.

## 4 Applications in precision medicine

### 4.1 DNA mutation

Genetic diagnosis (such as DNA mutations) for precise prevention and treatment presupposes the ability to accurately determine individual genetic information, thus genetic diagnosis is the foundation of precision medicine (Liu et al., 2020b). Current genetic testing is focused on tumor patients and rare single gene disease testing. In the future, as the expected cost of sequencing decreases from $500 in 2021 to $20 in 2030, genetic testing will become a common test item in diagnosis for more scenarios such as common diseases and medication guidance (Denny and Collins, 2021).

**FIGURE 5:**
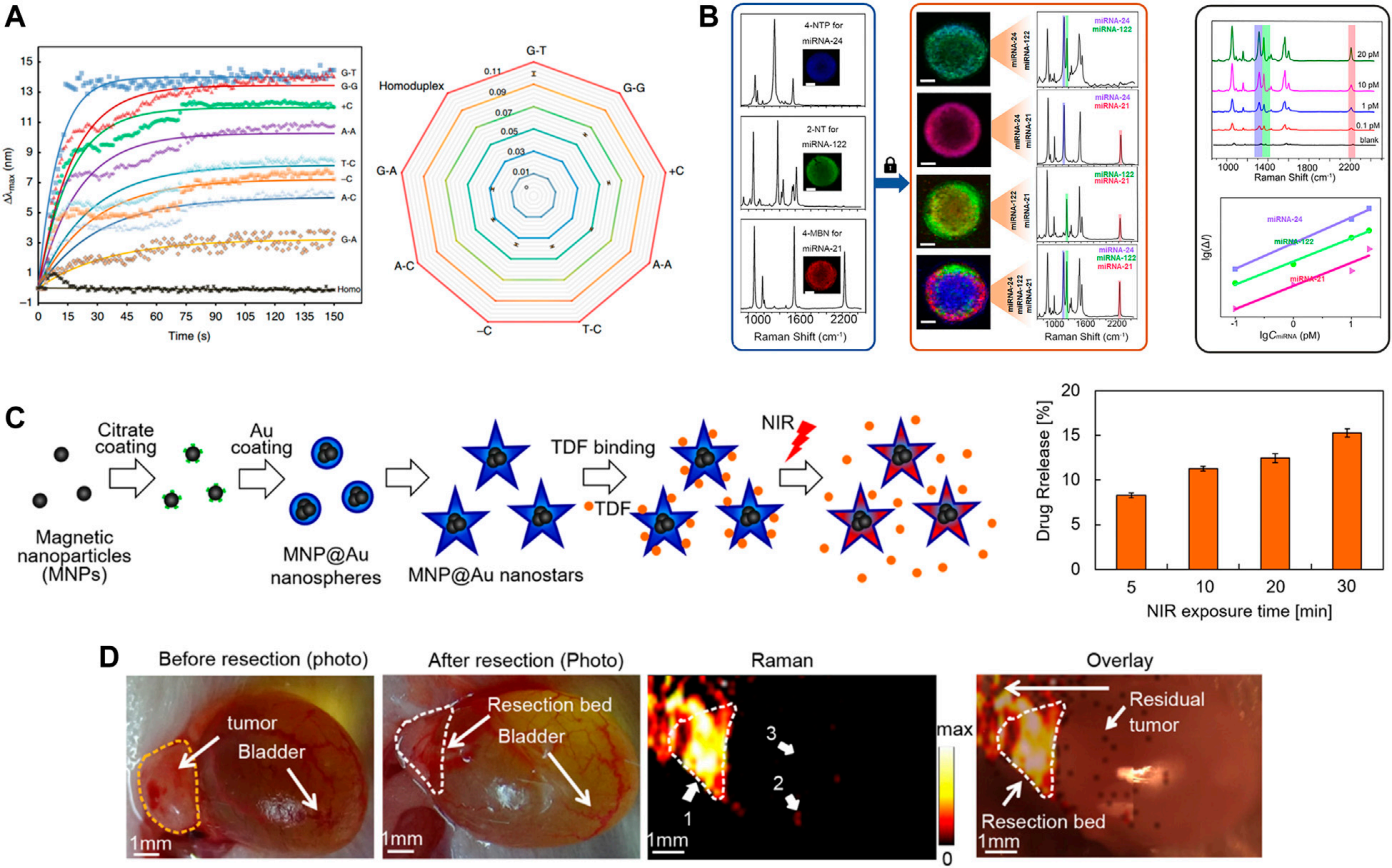
**(A)** Identifiable detection of the eight single point mutations. Reproduced with permission from (Ma et al., 2019). **(B)** Simultaneous detection of multiple miRNA targets. Reproduced with permission from (Lu et al., 2021). **(C)** Schematic diagram of MNP@Au nanostars synthesis steps, drug binding, and NIR-triggered drug release (left) and drug release after illumination with NIR. Reproduced with permission from (Tomitaka et al., 2020). **(D)** Intraoperative Raman imaging of residual microtumors after surgical resection of primary tumors. Reproduced with permission from (Qiu et al., 2018).

Scientists believe that identifying specific point mutations, deficiencies and nucleic acid modifications is becoming increasingly important as many sequences are confirmed as clinical biomarkers (Koch et al., 2018; Luo et al., 2018). Gene mutations are related to 10%–30% of spontaneous cancers in a diverse range of tissues (Hollstein et al., 2017). Most approaches to identifying gene mutations depend on traditional sequencing (Schmitz et al., 2018). However, these measures are still complex, which limits their use in the clinic. With the development of nanoplasmonic biosensors, researchers expect to solve this challenge through the design of rational plasmonic nanostructures. They effectively improve refractive index sensitivity, making them more sensitive than nano-plasmas of the same size (Ma et al., 2019). reported a rapid method for identifying point mutations through a AuNP sensor (Figure 5A). DNA-directed gold crystals form structurally designed rod-like nanoparticles with bridges based on the structure. Such plasmonic nanoparticles achieve a high refractive index to monitor trace amounts of protein-DNA binding without interference. Similarly (Liu et al., 2020b), proposed to combine active plasmonic nanostructures, SERS and polymerase chain reaction (PCR) to identify and classify BRAF wild type and V600E mutant genes using statistical tools. Among the four different shapes, the nanostar exhibited the highest SERS activity due to its highly anisotropic structure. Detection limits can be as low as 100 copies of the target DNA sequence.

In addition to high-precision detection of single-base mutations, because nanopores are sensitive to small changes in local refractive index, novel nanopore-based sequencing has been developed to open up new avenues for high-throughput nucleic acid sequencing (Garoli et al., 2019). Arif E. Cetin et al. describe a label-free sequencing platform integrated with a device based on lens-free imaging (Cetin et al., 2018). The spectral shift within the transmission resonance is triggered by a sharp decrease in the nanopore transmission response due to a mismatch in the spectral window upon attachment of streptavidin. This platform can reliably identify targeted deoxyribonucleic acids by monitoring plasma diffraction images. Nanopore sequencing will need to adapt the physical and chemical properties of solid-state nanopores and their compatibility with mass production in future developments to overcome the limitations of current electrical readouts and highlight potential advantages.

### 4.2 Cancer biomarker diagnosis

The diagnosis of cancer biomarkers is essential to achieve early disease prevention, monitoring progression and effectiveness of therapeutic interventions. Plasmonic sensors have demonstrated a wide array of analytical abilities, ranging from fast colorimetric readings generation to single molecule sensitivity at ultra-low sample volumes, which has allowed them to be increasingly explored in cancer biomarkers (Cathcart and Chen, 2020). A large number of biomarkers have been developed for cancer diagnosis, such as microRNA (miRNA), exosomes, circulating tumor DNA (ctDNA), and circulating tumor cells (CTCs) (Bellassai et al., 2019). For example, the presence of CTCs, whose phenotypic heterogeneity suggests different invasiveness, reveals the stage of cancer and metastasis (Poudineh et al., 2017). Among them, research on miRNA is progressing rapidly. There are now significant advances in miRNA applications of biosensors in breast, lung and colorectal cancers (Borghei and Hosseini, 2019; Wong et al., 2021; Azzouz et al., 2022; Ekiz Kanik et al., 2022). miRNA, a single-stranded non-coding RNA molecule of short length (usually about 18–25 nucleotides in length), have a critical role in apoptosis, proliferation, differentiation, invasion and migration of cells (Kappel and Keller, 2017). Therefore, miRNA is a potential and important indicator to distinguish benign and malignant tumors in certain contexts (Wong et al., 2021). However, miRNA detection is still complicated by the short length and low abundance of miRNAs and the high sequence similarity between members of the same family (D'Agata and Spoto, 2019). In principle, the operation of the plasma sensor relies on sensing changes in the local dielectric environment. miRNAs differing in sequence by only a single base are expected to show almost identical refractive indices, and thus should vary identically in the local dielectric environment.

Lu et al., 2021 reported the use of individual magnetic beads covered with a plasma layer as a multiplexed microreactor (Figure 5B). miRNA will result in the specific capture of the corresponding SERS reporter GNP being specifically captured into the epithelial layer, which will greatly boost SERS signal caused by the target miRNA. This signal will be mapped by confocal Raman microscopy. This method achieves high precision g-sensing of sub-pM targets with multiplex detection. Subsequently, attomolar-level assays were reported (Ekiz Kanik et al., 2022), developed a highly sensitive and multiplexed digital microarray using plasmonic Au nanorods as markers to enable high-precision two microRNA (miRNA-451a and miRNA-223-3p) detection. Particle tracking addresses the sensitivity constraints of biomarkers in the existence of low-affinity but high-abundance background molecules. Both miRNAs are 10 attomolar and the total incubation time is reduced from 5 h to 35 min.

### 4.3 Virus detection

The spread and proliferation of viruses has emerged as a risk to global biosecurity, with the current COVID-19 pandemic as an example. SARS-CoV-2 is an RNA virus that can be transmitted not only by inhalation of viral particles in droplets discharged into the air, but also by aerosols carrying viral droplets that can survive for up to 3 h, and by daily personal contact with contaminated surfaces (Lewis, 2020; Prather et al., 2020). The high level of antigenic drift of SARS-CoV-2 may allow the viral pathogen to find additional modes of transmission and become more lethal (Yewdell, 2021). With thousands of different types or variants of SARS-CoV-2 around the world, the variability of the virus underscores the urgent need to design effective vaccines, develop early and rapid diagnostics, and effective antiviral and protective therapies (Yakoubi and Dhafer, 2023). In this regard, the field of nanotechnology can be a bridge between diagnosis and treatment in the fight against COVID-19 and can provide many solutions both outside and inside the host.

Nanoplasmonic have antiviral activity against former coronaviruses and numerous other kinds of viruses, and by studying the photoelectric and chemical characteristics of plasmonic nanoparticles showing surface plasmon resonance effects, they may offer novel perspectives to combat COVID-19 as a drug carrier that is part of an effective therapy for early detection. On the one hand, it is based on the detection of metal surfaces. For example (Huang et al., 2021), developed a method for one-step rapid and direct optical measurement of SARS-CoV-2 virus particles using a spike-in protein-specific nanoplasmonic biosensor, which requires almost no sample preparation. Sensitivity down to 15 vp/mL. On the other hand, the detection is based on self-assembled metal structures. For example (Funari et al., 2020), developed a label-free microfluidic sensing platform with gold nanospikes fabricated by electrodeposition, where the concentration of the target antibody can be associated with the LSPR wavelength peak shift of the gold nanospikes due to local refractive index changes caused by antigen-antibody binding. The platform achieves a limit of detection of ∼0.08 ng/mL (∼0.5p.m.). To further improve sensitivity (Qiu et al., 2020), used complementary DNA receptor-functionalized two-dimensional gold nanoislands (AuNIs) that can accurately distinguish selected sequences of SARS-CoV-2 by nucleic acid hybridization with a low detection limit of 0.22 p.m.

In terms of external conditions, existing personal protective equipment can actually be an effective measure to restrain the spreading of SARS-CoV-2, but there is no inherent antimicrobial effect can only temporarily protect the user (Yakoubi and Dhafer, 2023). Therefore, the development of anti-viral surface coatings and self-sterilizing surfaces to inactivate SAS-CoV-2 is an issue of high demand. In these respects, several researches have recently been presented highlighting the use of plasma metal nanoparticles in combination with polymers and textiles that play a role in decreasing the survival of viruses on the surface (Toledo et al., 2020; Ulucan-Karnak, 2021). Ordinary masks and N95 respirators usually lack self-sterilizing properties, and water droplets can still remain on the fibers (Zhong et al., 2020). reported plasma photothermal and superhydrophobic coatings on N95 respirators, where the superhydrophobic properties prevent the accumulation of respiratory droplets on the respirator surface and the presence of silver nanoparticles provides additional protection against microorganisms through the disinfection of silver ions. The plasmonic heating can raise the respirator surface temperature to more than 1 °C within 80 min of sunlight exposure. This approach offers greater long-term protection by enhancing the reusability and antimicrobial activity of the mask.

### 4.4 Evaluation of vaccine effectiveness

Although the COVID-19 vaccine has significantly transformed the struggle against pandemics, for many people there is hesitation to get vaccinated (Fischer et al., 2022). Detection of vaccines (adjuvants and antigens) is therefore critical for basic research in immunotherapy and may influence the extent to which people accommodate vaccines in the future (Shen et al., 2021). Meanwhile, the development of antibody assays is essential for monitoring and epidemic studies, evaluating the level and persistence of antibodies required for immunization, and assessing vaccine effectiveness. There appears to be less information on the evaluation of nanoplasmonic biosensors in terms of vaccine efficacy, probably because of difficulties such as assessing antibody maturation and distinguishing recent from old infections, but this is still very important.

Park et al., 2021 performed LSPR to evaluate real-time adsorption of bovine serum albumin (BSA) on alumina and silica surfaces using alumina- and silica-coated silver nanodiscs arrays with plasmonic properties. A more rigidly adherent BSA protein-based coating was formed on the surface of alumina-based nanomaterials. Helps guide the development of protein coatings for vaccines. Antibody and antibody affinity assays are also among the uses of plasma in vaccines (Liu et al., 2020a). simultaneously detected antibodies to the spindle S1 subunit and SARS-CoV-2 receptor binding domain in human serum and saliva by near-infrared fluorescence amplification of proton Au substrates, and quantified immunoglobulin affinities against coronavirus antigens from SARS-CoV-2, SARS-CoV-1, and common cold virus. The effectiveness and efficacy of most studies involving vaccines against mutant strains is unknown. In addition, the longer COVID-19 persists, the more likely new mutations which assist the virus to escape the immune response will appear. Therefore, further studies on different types of vaccines and cross-vaccination are needed.

### 4.5 Drug delivery

Due to the expanding imaging and diagnostic capabilities of nanoplasmonic biosensors in response to exterior stimulation, they are already being explored for on-demand drug detection (Asadishad et al., 2021), drug resistance analysis (Ouyang et al., 2023), drug attrition reduction (Lee et al., 2021), drug delivery (Figure 5C) (Tomitaka et al., 2020) and toxicological analysis (Masterson et al., 2021). Due to individual differences, such as medication history, age, and other factors, routine doses of medications may not be appropriate, which may lead to organ damage or other complications for the patient.

The number of nanomedicines currently available to patients is much lower than predicted by the field, in part because of the translational gap between animal and human studies (Mitragotri et al., 2017). Overcoming the patients’ heterogeneous biological, microenvironmental and cellular barriers is also achieved through precision therapy (Mitchell et al., 2021). As a result, few of the approved nanomedicines are suggested as preferred treatments and many improve the condition of only a small percentage of patients. However, nanoplasmonic development can begin to optimize drug delivery in a more individualized way and enter the era of precision medicine. sweat usually contains large amounts of biochemical substances (such as electrolytes, metabolites, proteins, and drugs), and can reflect human physiological conditions (Xiao et al., 2023). achieved simultaneous monitoring of vital signs with sweat sampling and acetaminophen drugs. Some treatments with nanoplasmonic are intended to facilitate particle build-up and penetration by reshaping the tumor microenvironment, thereby improving drug efficacy or sensitizing tumors to specific therapies. For example (Chen et al., 2019), used photothermal NPs to improve the infiltration and activity of chimeric antigen receptor (CAR) T cells against solid tumors. Similarly (Wilson et al., 2016), reported that tumor-associated endothelial cells can be manipulated by microRNAs delivered by NPs that alter the tumor vascular system, thereby sensitizing the tumor to conventional cancer therapies.

In conclusion, the introduction of nanoplasmonic developed for specific patient populations can accelerate clinical translation. Advances in genome sequencing and biomarker diagnostics permit suitable choices for the treatment of patient-specific diseases, as mentioned earlier. The development of drug delivery for precision medicine should be a highly customizable process. Such well-designed methods enable the pharmacokinetics of therapeutic agents to be tuned to meet solubility, delivery or biodistribution requirements.

### 4.6 Molecular imaging

Nanoplasmonic in SPR and LSPR imaging are applied to microscopic imaging techniques in biology and biochemistry to improve key properties of fluorescence imaging (such as sensitivity and resolution) (Ahn et al., 2021). SPR and LSPR imaging are actively used in studies to monitor changes in surface properties with specific markers for functionalized imaging (such as DNA fixation/hybridization processes and antibody-antigen interactions) (Aoki et al., 2019; Zhou et al., 2019). In recent studies, improvements in nanoplasmonic element-based fluorescence imaging have reached the level of super-resolution imaging, pushing the diffraction limit (Brettin et al., 2019). For example (Son et al., 2019), demonstrated that hyperlocalized near fields with plasmonic nanopore arrays (PNA) studied neuronal mitochondrial transport. Compared to conventional imaging techniques, PNAs create large arrays of hyperlocalized beams and allow sampling and extraction of 3D mitochondrial dynamics in almost real time. The resolution is improved by a factor of 12.7 compared to confocal fluorescence microscopy.

SERS-based nanoprobes are used as competitive imaging agents for *in vitro* and *in vivo* bioimaging due to their ultra-sensitivity, specificity, multiplicity, biocompatibility and photostability (Lin et al., 2021b). In addition to its use in cancer diagnosis, SERS has shown an increasingly important role in cancer treatment. On the one hand, there is growing evidence that SERS has become a new imaging tool to guide surgeons in pinpointing the margins of surgically removed tumors. On the other hand, there has been considerable interest in smart SERS-based therapeutic diagnostic platforms for SERS guidance. For example (Qiu et al., 2018), demonstrated intraoperative detection and eradication of residual microscopic lesions at the surgical margins, which relies on gap-enhanced Raman tags (GERTs)-based Raman imaging (Figure 5D). The low energy laser at 785 nm triggers a Raman signal that can be used for highly sensitive and photostable detection of microtumors. And the thermal effect of microtumor ablation is produced when switching between 808 nm high power lasers. However, since the expression of biomarkers in tumors is heterogeneous, attention should be paid to the issue of multiple molecular imaging of different disease-associated biomarkers.

## 5 Outlook and conclusion

Nanoplasmonic, a promising nanomaterial, has unique plasmonic and optical properties including absorption, scattering, and field enhancement. In this review, we disclose that nanoplasmonic biosensors have great potential for applications in precision medicine. The first part demonstrates specific sensing based on nanoplasmonic (such as SPR, LSPR and SERS) and analyzes their existing advantages and shortcomings. The second part reports on the integration of multiple technologies, including integrated isothermal amplification, CRISPR/Cas, lab on a chip and ELISA, which help to bridge the gaps of individual technologies. Based on the current situation, POC testing is more appropriate for these sensors than large-scale testing, although micro and portable sensors are appearing. In the final section we discuss the applications and trends of nanoplasmonic biosensors in precision medicine. In particular, the continuing COVID-19 pandemic has highlighted the need to find fast and reliable sensors. In fact, in a sense, virus pandemics and insurmountable cancers are driving the field of precision medicine sensing, especially plasmonic technology, as the requirement for rapid, dependable, mobile and inexpensive sensors becomes essential.

Despite the rapid development of nanoplasmonic with controlled optical properties, their large-scale utilization in medicine is still limited, probably due to i) the relatively high price of precious metals, ii) the low yield of materials with excellent properties, iii) the need for precise fabrication methods to control different sizes and morphologies, iv) the unclear biological toxicity in physiological environments, and v) the complex matrix identification capabilities in complex matrices. In most applications, the optical properties of nanoplasmonic are strongly dependent on size, morphology, and interactions with each other, so care must be taken to adapt fabrication techniques to achieve well-controlled and highly productive plasmas with various structures. In order to be stable in maintaining optical properties, polymers and inorganic coatings have been successfully used with many effective surface modification strategies that contribute to chemical and biological stability. However, issues such as long-term biocompatibility and specific cytotoxic residues of nanoplasmonic under pathological conditions remain a challenge for clinical practice.

Several issues need to be addressed in nanoplasmonic integration technology. The first is the integration of nanoplasmonic biosensors with sample pre-processing units (such as separation and purification of samples) for eventual practical applications in POC as well as cost-effective sensors for mass production, and more ideally will be used to deliver advanced nanoplasmonic technology from the laboratory to the bedside. As a result, the need for plasma coupled to microfluidics for sample collection and processing will continue to grow. With development it can be expected that mobile and economical biosensors will be available. Secondly, while nanoplasmonic biosensors are very promising for detecting the redox activity of individual proteins or enzymes, they still face challenges due to the limitations of current amplification strategies. The application of nanopores will not only be applied to commercial DNA sequencing, but will also contribute to the study of protein binding and enzyme reaction kinetics.

In conclusion, nanoplasmonic biosensors have achieved significant advances and have shown sufficient sensitivity to observe even single molecular binding events. The quantification of biomolecular interactions at the single molecule level greatly expands the scope of biosensor and analytical techniques and allows access to potential heterogeneity in molecular properties. This heterogeneity may arise from the existence of various species in the sample or the presence of distinct conformations of the same species. However, transforming these encouraging advances in science to biosensor devices for daily life will require additional work on many aspects. The next-generation of nanoplasmonic technology will move out of the laboratory and translate into smart POC diagnostics that will shape the future of precision medicine.
